# Standardization of DNA extraction from paraffinized spleen samples: molecular diagnosis of human malaria

**DOI:** 10.1186/s12936-023-04764-3

**Published:** 2023-11-27

**Authors:** Raimunda Sandra Pacheco Souza, Monique F. dos Reis, Luiz Carlos de Lima Ferreira, Manuela C. Morais, Antonio Kassio S. Lima, Laila Rowena Albuquerque Barbosa, Gisely Cardoso de Melo, Marcus Vinicius Guimaraes de Lacerda

**Affiliations:** 1 Leonidas & Maria Deane Institute (ILMD), Fiocruz, Manaus, Amazonas 69057-070 Brazil; 2 Federal University of Amazonas, Manaus, Amazonas 69080-900 Brazil; 3 Oncology Foundation (FCECON), Manaus, Amazonas 69040-010 Brazil; 4 Amazonas State University (UEA), Manaus, Amazonas 69040-000 Brazil; 5 Doctor Heitor Vieira Dourado Tropical Medicine Foundation, Manaus, Amazonas 69040-000 Brazil

## Abstract

**Background:**

*Plasmodium vivax* is the main species responsible for human malaria in Brazil, and one of its manifestations is splenic malaria, though there are still challenges in its diagnosis. The present study aimed to standardize *Plasmodium* sp. DNA extraction from histological slices of spleen and diagnosis using real-time qPCR.

**Methods:**

This study performed a microtomy of a paraffin-embedded spleen as a positive control for *P. vivax* from a patient who had been previously diagnosed with the parasite. The sample was deparaffinized with xylol and ethanol, then DNA extraction was performed with two commercial kits. qPCR was carried out with the *Taqman* system for detection of *Plasmodium* sp. and was made species-specific using *PvmtCOX1* gene. From 2015 to 2019, 200 spleen samples were obtained from trauma patients subjected to splenectomy in Manaus, Amazonas. All the samples were tested for cell-free human DNA (cfDNA).

**Results:**

The deparaffinization and the *Plasmodium vivax* DNA extraction method was successfully standardized, and the control sample was positive for *P. vivax.* Of the 200 samples, all qPCRs were negative, but they were positive for human PCR.

**Conclusion:**

Paraffinization is practical and efficient for the preservation of samples, but the formation of bonds between proteins and DNA makes extraction difficult. Despite this, in this study, it was possible to standardize a method of DNA extraction for detecting *P. vivax.*

## Background

Due to its high rates of morbidity and mortality, malaria is one of the main public health problems in the world. It is an infectious disease caused by *Plasmodium* spp. and is transmitted by the bite of the female *Anopheles* mosquito [1]. In Brazil, most cases of malaria occur in its Amazon region and approximately 82% of cases are caused by *Plasmodium vivax* [2].

Some patients do not present febrile paroxysm, being called asymptomatic carriers, which may contribute to the transmission of malaria due to the lack of sensitive diagnosis and therapeutic intervention [3]. The biomass of asexual parasites can accumulate in the spleen of asymptomatic humans infected with *P. vivax.* It is assumed that reticulocytes, in which *P. vivax* develops, may present high densities in the spleen, providing a niche for the survival of the parasite [4]. Then, molecular methods are very useful in detecting genetic material of parasites, thus allowing the identification and differentiation of species in cases of low parasitaemia and asymptomatic cases [5].

DNA extracted from tissues embedded in paraffin can be used for diagnosis of malaria using PCR, and there are even studies based on analysis of genomic or mitochondrial sequences of this type of samples [6]. Funabashi and Iwamura [7] showed that the QIAGEN kit is able to produce a high degree of purity, and reproducible and consistent results in PCR amplification of β-actin and AMEL genes and in short tandem repeat (STR) analysis.

The state of Amazonas is endemic for malaria and has a large number of infections with low parasitaemia, which are often not diagnosed [8]. There is a hypothesis that the parasites accumulate in the spleen of infected people. Therefore, the present study aimed to standardize DNA extraction from paraffinized spleens for the detection of *Plasmodium* spp. using qPCR.

The methods validated in this study may be important for pathological investigations involving formalin-fixed, paraffin-embedded (FFPE) samples. This is relevant because most samples are available in this format, and validation of these methods with FFPE could expand their clinical and research applicability. Application of the developed methodology makes it possible to detect malaria parasites in spleens, contributes to scientific research, playing an important role in the fight against malaria, as splenic rupture can occur more frequently in these regions. The density of malaria parasites in an FFPE sample can be very different from the abundance of target genes in other diseases. Because each disease has specific characteristics that can influence the choice of analysis methods. Therefore, it is suggested that detection methods be validated for each investigation that is intended to be carried out.

## Methods

### Control samples

This study was developed at Fundação de Medicina Tropical Dr. Heitor Vieira Dourado (FMT-HVD) in Manaus, Amazonas. A paraffinized spleen from a patient previously diagnosed with *P. vivax* was used as the control [9]. As an inclusion criterion, the block should be in a viable condition for use, have been adequately stored, and there should be enough material to carry out the analyses.

### Histological sections

Samples containing five, six, eight, ten, fifteen, twenty and twenty-five slices with a thickness of 10 μm each and samples containing five and ten slices with a thickness of 20 μm were prepared. The largest axis of the block was placed vertically to the knife edge, then the paraffin was removed from the block. The block chilled on ice to moisten the paraffin, and, after cooling, cuts were made using a scalpel. The cuts were placed in microtubes.

### Deparaffinization

For the samples containing 5, 10, 15, 20 slices, washes were performed with xylene (1 mL), centrifugation for 5 min at 14,000 rpm, washing with 1 mL of absolute alcohol and centrifugation for 5 min at 14,000 rpm, with the supernatant being subsequently discarded. Then, the samples were washed again with 70% alcohol and left to dry. Twenty-five samples were washed with 1 ml of xylene, centrifuged for 5 min at 14,000 rpm, then washed with absolute alcohol, and dried at room temperature. The rest of the sections were deparaffinized with two washes of xylene and two washes of absolute alcohol for each wash, being centrifuged for 5 min at 14,000 rpm and the supernatant subsequently discarded. All samples were dried and then weighed in milligrams.

### DNA extraction

Extraction was performed with two QIAGEN extraction kits. For the QIAamp FAST DNA Tissue kit, 200 µL of Buffer AVE, 40 µL of Buffer VXL, and 1 µL of Buffer DX were added to the sample. This was then incubated in a water bath at 37 °C for 6–8 h and, following this, incubated in a thermomixer for 12 h. After the incubation period, 20 µL of Proteinase K and 4 µL of RNAase were added, homogenized in vortex, and left at room temperature for 72 h. Afterwards, the mixture was carefully transferred to the filter tube provided by the manufacturer and, thereafter, the manufacturer’s instructions were followed.

For the QIAamp DNA Blood Minikit, 20 µL of proteinase K were added to the sample and vortexed. This was then incubated for 6 to 8 h in a water bath at 37 °C, then 200 µL of Buffer AL were added and the mixture was left at room temperature for 72 h. Subsequently, 200 µL of ethanol (95–100%) were added and the mixture was vortexed immediately. Bubbles were removed by centrifugation and the mixture was carefully transferred to the filter tube. Thereafter, the manufacturer’s instructions were followed.

DNA quantification in g/µL was performed using spectrophotometry (NANODROP). Values above 10 ng/µL were considered acceptable.

### qPCR

qPCR was carried out with the Taqman system for detection of *Plasmodium* sp. and was species-specific using *PvmtCOX1* gene [JD1] (Table 1) [10, 11]. All samples were tested in triplicate, the volume was 24 µL including the reagent mix and the genetic material, with a 10 µM concentration of primers and probes. For the quantification of the number of copies plasmids containing the gene fragment were used, with the following three dilutions: 10^2^, 10^4^ and 10^6^ copies/µL, and a negative control was also used. Amplification and qPCR measurements were performed using the Applied Biosystems 7500 Fast Real-Time PCR system.

**Table 1:** Primers and probes used for amplification via qPCR. Source: [10, 21, 22]

Assay	Primers	Sequence 5′–3′
QMAL	Forward	TTA GAT TGC TTC CTT CAG TRC CTT ATG
	Reverse	TGT TGA GTC AAA TTA AGC CGC AA
	Probe	6FAM—TCA ATT CTT TTA ACT TTC TCG CTT GCG CGA—BHQ1
*PvmtCOX1*	PvMt_cox1_Fw	TTATATCCACCATTAAGTACATCACTT
	PvMt_cox1_Rev	AACCTTTAGATCTTAGATGCATTACA
	PvMt_cox1_Probe	FAM-CCTGTTGCAGTAGATGTTATCATTG-BHQ1
*cfDNA*	For	GGC ACA CGT GGC TTT TCG
	Rev	GGT GAA CCT CGT AAG TTT ATG CAA
	Probe	VIC5-TCA GGA CGT CGA GTG GAC ACG GTG-3 TAMRA

### Selection of samples for testing

From 2015 to 2019, samples of spleens in paraffin blocks were selected from a reference laboratory for histopathological examination in Manaus, Amazonas. The selected samples were from individual trauma victims submitted to splenectomy. The samples came from patients of any age and of either sex. Samples with adequate storage and at room temperature, with enough material to obtain the cuts were included.

### Microtomy, deparaffinization extraction and qPCR of test samples

Ten slices with a thickness of 20 µM were made, which were deparaffinized with two washes of xylol and two washes of ethanol. At each wash, centrifugation was performed for 5 min at 14,000 rpm and the supernatant was discarded. The methodology established by the manufacturer for the QIAamp FAST DNA Tissue kit and QIAamp DNA Blood Minikit were adapted for this study. In qPCR, the samples were tested for specific genus (QMAL) and species of *P. vivax.* qPCR for human DNA was also performed (*cfDNA* gene) (Table 1) [12].

## Results

Table 2 shows the difference in the number of tissue slices and their thickness, as well as the washes performed, and results of qPCR performed. In addition, 1–2 washes with xylol and ethanol were adapted for deparaffinization. It was observed that the best result was with ten cuts of 20 μm, two washes of xylol and two washes of absolute alcohol, weighing 40 mg. The DNA concentration of 50 ng/µL was obtained using a QIAamp DNA Blood Minikit and 55 ng/µL was obtained using a QIAamp FAST DNA Tissue Kit, with a positive qPCR result for the specific genus and species in the control sample.

**Table 2:** Results of the steps of deparaffinization, weight, DNA concentration and qPCR with copy number of tissue sections

Slices/thickness	Wash xylol	Wash—absolute alcohol	Wash—alcohol 70%	Dry weight (mg)	DNA (Blood Kit), ng/µL	DNA (Tissue Kit), ng/µL	PCR *Plasmodium* sp	PCR *Plasmodium vivax*	Copy number *Plasmodium* 1 (copies/µL)	Copy number *Plasmodium* 2 (copies/µL)	Copy number *P. vivax* 1 (copies/µL)	Copy number *P. vivax* 2 (copies/µL)
5/10 µm	One 1′	One 1′	One 1′	70	5	8	Negative	Negative	0	0	0	0
10/10 µm	One 1′	One 1′	One 1′	84.8	9	7	Negative	Negative	0	0	0	0
15/10 µm	One 1′	One 1′	One 1′	125	2	15	Negative	Negative	0	0	0	0
20/10 µm	One 1′	One 1′	One 1′	122.3	15	20	Negative	Negative	0	0	0	0
25/10 µm	One 1′	One 1′		177	47	32	Negative	Negative	0	0	0	0
6/10 µm	Two 1′	Two 1′		62	61.8	70	Negative	Negative	0	0	0	0
8/10 µm	Two 1′	Two 1′		41	35	41	Negative	Negative	0	0	0	0
5/20 µm	Two 1′	Two 1′		23	40	32	Negative	Negative	0	0	0	0
10/20 µm	Two 1′	Two 1′		40	50	55	Positive	Positive	75.01 [73.8–76.4]	86.03 [84.2–87.9]	89.2 [87.4–91.1]	106.06 [105.2–108]

DNA concentration obtained per ng/µL (DNA). Number of samples selected in qPCR for samples taken with the sample collection kit (copy number *Plasmodium* 1), number of copies obtained via qPCR for samples using the tissue kit (copy number *Plasmodium* 2), number of samples obtained via qPCR for samples using blood kit (copy number *P. vivax* 1), number of copies obtained via qPCR for samples using the tissue kit (copy number *P. vivax*). Confidence interval shown in square brackets [CI]

**Fig. 1:**
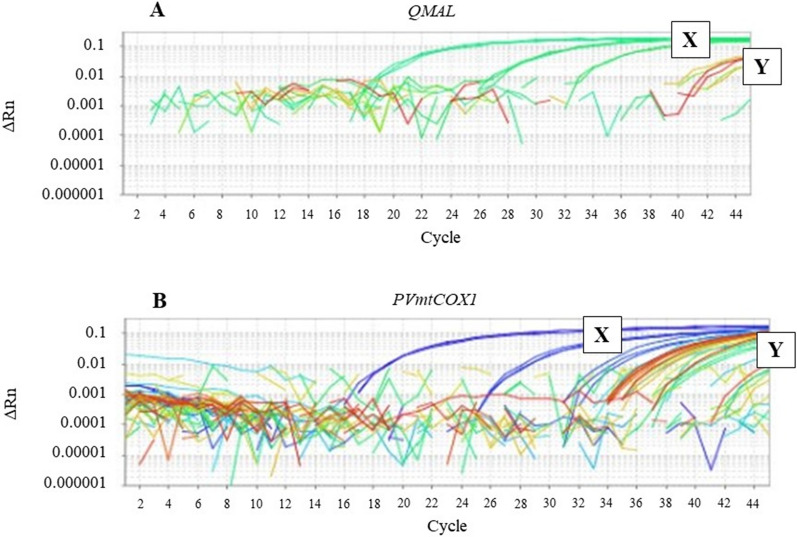
qPCR curves for control sample. **a** QMAL qPCR result; X—DNA samples from spleens; Y—Amplification of plasmids **b** *PVMTCOX1* qPCR result; X—DNA samples from spleens; Y—Amplification of plasmids

Figure 1 shows the results for the positive control sample. The copy number for Qmal was 75.01 copies/µL (73.8–76.4) using the blood kit and 86.03 copies/µL (84.2–87.9) for the tissue extraction kit. For *PvMTCOX1*, the sample extracted with the blood kit had a mean copy number of 89.2 copies/µL (CI 87.4–91.1) and, with the tissue kit, it had a mean of 106.06 copies/µL (CI 105.2–108).

For the two hundred samples that were analysed, all were negative for malaria. However, they were observed to be positive for human DNA (cfDNA), with a copy number of 105.02 copies/µL (CI 103.1–107).

## Discussion

The spleen may play an important role in malaria, with the presence of haematomas, splenic infarctions, ruptures and abscess formation [13]. According to Mohamed et al. [14], most cases of splenic rupture were caused by *P. vivax* and *Plasmodium falciparum*. The spleen’s participation in parasite loads is reinforced by high parasitaemias and clinical severity in splenectomy patients [15]. Siqueira et al. [16] described a patient with *P. vivax* detected in the blood after undergoing a splenectomy due to a spleen rupture.

Lacerda et al. [9] used twenty 10 μm paraffin tissue sections from the spleen, brain and lung for DNA analysis and a nested PCR was performed using 200 ng of DNA to confirm *P. vivax* infection. The presence of *P. vivax* was detected in the spleen using this method.

Kho et al. [4] standardized DNA extraction from frozen biopsy specimens. The present study was standardized on tissue samples embedded in paraffin blocks; however, some factors can influence the quality of the DNA in paraffin blocks, such as length of the prefixation period, fixation and decalcification [17]. The difficulty of accessing and processing these samples remains a challenge, but quality was possible and suitable for analyses, such as DNA extraction and detection of *Plasmodium* spp. and human DNA by qPCR. This was possible due to the appropriate handling of these samples, thus contributing to important advances in the understanding of malaria. PCR performed using genetic material extracted from a paraffin-embedded sample is widely used; however, standardization must always be carried out to suit the amplification conditions. For this type of material, special attention must be paid to factors that are endogenous and exogenous to the reaction, starting with the fixation methods up to the shelf life of the paraffin block [18].

Elgayoum et al. [19] performed a qPCR validation for splenomegaly related to malarial infection that used the thick blood smear and qPCR in peripheral blood and related it to the size of the spleen. In this present study, qPCR was performed directly on spleen samples, showing that the parasite can in fact be present in the spleen and can be detected by a highly specific method.

Due to the unavailability of fresh spleens in this study, the study emphasized the importance of the applicability of the method developed in FFPE samples, making it very promising in genomic and transcriptomic analyses. Despite this, this work observed that all spleen samples from trauma victims were positive for human-targeted PCR, which demonstrates that the deparaffinization and DNA extraction process was satisfactory. Elizalde-Torrent et al. [20] described a splenic rupture caused by *P. vivax*, which was identified using ultrasound, electron microscopy and conventional PCR. There are still few studies focused on methodologies for diagnosing malaria in the paraffinized spleen, most of which are focused on frozen samples and PCR of peripheral blood. Here, this study showed an optimized methodology that employs deparaffinization with xylol and ethanol, which is necessary to obtain DNA, and that qPCR is possible without previous DNA purification.

## Conclusion

In this work standardized the deparaffinization and DNA extraction of samples of paraffinized spleens and the detection of *P. vivax* DNA. The contribution of this study was to elucidate and minimize the problems involved in using paraffinized histological samples for DNA extraction and analysis.

## Data Availability

Datasets from the current study are available upon reasonable request to the corresponding author.
